# Fetal Cardiac Collapse Diagnosed By Umbilical Venous Flow Volume After Thoraco-Amniotic Shunting for Severe Pleural Effusion

**DOI:** 10.1055/a-2697-2212

**Published:** 2025-09-16

**Authors:** Yuichiro Takahashi, Shigenori Iwagaki, Kazuhiko Asai, Masako Matsui, Ryuichi Shimaoka, Hitomi Ono, Saki Inuzuka

**Affiliations:** 1 Department of Fetal-Maternal Medicine, Obstetrics, Gifu Prefectural General Medical Center, Gifu City, Gifu, Japan; 2 Department of Fetal-Maternal Medicine, Nagara Medical Center, Gifu, Japan

**Keywords:** thoraco-amniotic shunting, fetal pleural effusion, hydrops fetalis, cardiac collapse, cardiac failure, UVFV, chylothorax, Frank–Starling's law

## Abstract

**Objective:**

Although thoraco-amniotic shunting (TAS) for severe pleural effusion is an effective fetal treatment, there are some cases in which it deteriorates, showing circulatory collapse. To evaluate the usefulness of umbilical venous blood flow volume (UVFV) for predicting deterioration, we analyzed the fetal low UVFV situation.

**Methods:**

In 22 cases of fetal severe pleural effusion, we measured UVFV/fetal estimated birth weight (mL/minute/kg) prospectively before and after TAS by ultrasonography. We defined low UVFV/kg as < 50 mL/minute/kg (2.5 percentile) and compared subgroups based on their UVFV value and analyzed the outcome after birth.

**Results:**

Total survival rate was 59% at 6 months. Seven cases in the low group before delivery (UVFV/kg 19.5) showed poor prognoses, such as fetal/neonatal death and longer neonatal intensive care unit management (100% vs. the normal UVFV group 40%, *p* = 0.017). The low group also showed umbilical artery absent end-diastolic velocity (71%); edema resolved in 50%, suggesting hypo inflow from the placenta and fetal hypocardiac output status, revealing fetal cardiac collapse.

**Conclusion:**

UVFV analyses would be a new marker of fetal management of severe pleural effusion, suggesting low UVFV after TAS seems to be hypovolemic cardiac collapse and shows poor prognosis, and we had better consider immediate delivery to prevent death even after TAS.

In recent years, thoraco-amniotic shunting (TAS) for fetal pleural effusion has been conducted as a fetal therapy. 1 2 3 4 5 6 In particular, it has been effective for cases complicated with fetal hydrops. Recent studies have reported on the effectiveness of TAS, with reported survival rates of 61 to 70%. 3 4 5 6 7 8 However, some cases have a poor prognosis even if the fetal edema resolves. Therefore, the next step of this therapy should be a further and innovative evaluation of fetal cardiac status, as well as the development of new criteria for the limitations of TAS and indications for immediate delivery that can prevent fetal death even after TAS. Since fetal cardiac function using ultrasonography has not been elucidated to date, we attempted to clarify fetal cardiac function with a particular focus on fetal preload, judging from umbilical venous blood flow volume (UVFV) 9 10 11 as markers of hypovolemic status caused by lymphatic drainage from the fetus to the amniotic cavity. Our hypothesis is that, as Frank–Starling's law says, if the fetal preload reduces too much because of reduced UVFV, fetal heart failure, such as contractility, would be affected. So, we examined UVFV before and after TAS and developed a new classification of fetal deterioration by using these parameters; in addition, we report the various patterns of fetal circulation at different stages of collapse of this disease.

## Methods

This is a retrospective cohort study to evaluate fetal circulatory parameters before and after TAS for severe fetal pleural effusion. We performed TAS on the fetuses with re-pooled massive pleural effusions after thoracocentesis within 7 days. We excluded cases with fatal major chromosomal abnormalities and structural anomalies. TAS was performed using a double-basket catheter (Hakko, Nagano, Japan) 6 7 under local anesthesia. After TAS, routine obstetrical management was initiated; tocolysis and amnioreduction were performed if indicated. The decision to deliver was dependent on the usual obstetrical management. All infants were managed in the neonatal intensive care unit (NICU) with a high level of supportive care, such as respiratory management with high-frequency oscillation, intermittent mandatory ventilation, and chest drainage.

**Fig. 1:**
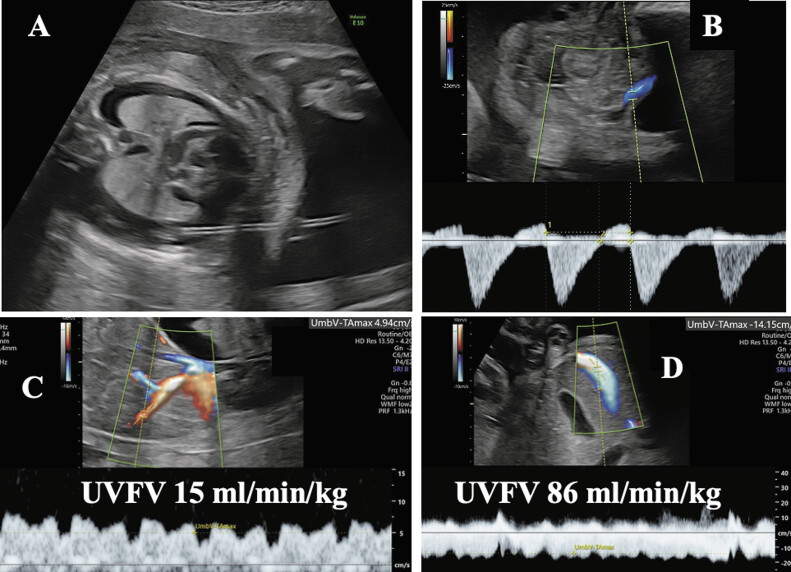
( **A** ) B-mode ultrasonographic image of fetus after thoraco-amniotic shunting (TAS) at 27 ^2/7^ weeks. ( **B** ) Color-Doppler image of pelvic umbilical artery-end diastolic reverse flow before TAS. ( **C** ) Pulse-Doppler image of umbilical venous flow volume (UVFV) before TAS, who showed cardiac collapse diagnosed by low UVFV 15 mL/minute/kg (<50 mL/minute/kg). ( **D** ) Pulse-Doppler image of UVFV after TAS, who showed improvement from collapse diagnosed by normal UVFV 86 mL/minute/kg (>50 mL/minute/kg).

Before and after TAS, the following ultrasonographic parameters were measured: biometry, amniotic fluid volume, cardio-thoracic area ratio (CTAR), skin edema, umbilical artery (UA) Doppler, middle-cerebral artery (MCA) Doppler, descending aorta maximum velocity (DA- *V*_max_ ), 12 ductus venosus (DV) Doppler, and UVFV preoperative within 2 days and postoperative weekly follow-up. For UVFV, several methods have been employed based on the area of measurement. We preferred previously reported methods 10 11 13 14 15 and selected the intra-hepatic portion for calculation. UVFV was calculated by the formulas: 0.5 × time-averaged maximum velocity × π × (UV diameter/2) 2 when using Voluson E8 and E10 (GE Ultrasound, Waukesha, Wisconsin, United States). Measurements were repeated two to three times, and their average value was used for analysis. The angles were maintained within 45 degrees in the measuring vessels. ( Fig. 1 )

Absolute normal UVFV has been discussed in recent years; we defined average UVFV as 110 mL/minute/kg and low UVFV as 50 mL/minute/kg at the 2.5 percentile, as previously reported. 10 For estimation of the actual fetal body weight, less the added weight of hydrops at birth, we got the standard deviation from the biparietal diameter and calculated the estimated fetal body weight using the Z score of BPD, excluding edema. This method could result in the determination of the “ideal fetal body weight” that excludes edema.

This protocol was approved by the ethical committee of our institution, Nagara-Medical Center, and Gifu Prefectural General Medical Center, and informed consent was obtained from all patients.

## Results

A total of 22 patients were enrolled and underwent TAS. Total survival rate at 28 days of age was 64%; it was 59% at 12 months. Median UVFV/kg (mL/minute/kg) before TAS was 89 (20–309), and final UVFV/kg before delivery was 92 (11–405).

In 68% of the cases, skin edema decreased; moreover, in 50% of the cases, it disappeared completely. Profiles and prognoses of all cases with hypo UVFV (<50 mL/minute/kg) at least one time during the management are described in Tables 1 and 2 .

**Table 1:** Cases profiles of 22 TAS cases

TAS ( *n* )	22
Hydrops before TAS	20 (91%)
TAS GA (wk)	29.2 (± 2.8)
Decreased edema after TAS	15/22 (68%)
Delivery GA (wk)	32.6 (± 3.1)
Birth weight (g)	1,933 (± 639)
UVFV/kg (mL/min/kg) before TAS	89 (20–309)
UVFV/kg (mL/min/kg) before birth	92 (11–405)
Survival at 28 d	14/22 (64%)
Total survival (6 mo)	13/22 (59%)

Abbreviationsreprinted: ± , mean ± SD; (−), median and its range; TAS, thoraco-amniotic shunting; UVFV, umbilical venous flow volume.

**Table 2:** Profiles and prognoses of all cases with one or more episodes of low umbilical venous flow volume (<50 mL/minute/kg) that exhibited pleural effusion before and after thoraco-amniotic shunting

Case	TAS wk	Before TAS	Before TAS UVFV/kg	Minimum UVFV/kg	Minimum UVFV post-TAS date(d)	Improved UVFV value (d)	Before birth UVFV/kg	Edema	UA-AEDV at low UVFV	Birth wk	Birth weight	Prognosis
		Edema						After TAS			(g)	
1	26	+	106	16	24	Not improved	16	−	+	31	1,130	FD
2	33	+	75	21	6	Not improved	21	+	+	33	1,474	ND
3	28	+	162	15	38	Not improved	15	+	−	33	1,793	ND
4	24	+	97	27	27	Not improved	27	−	+	27	1,066	ND
5	31	+	40	20	4	Not improved	20	−	+	32	1,680	Alive, NICU
6	30	+	37	11	2	Not improved	11	+	+	30	1,079	Alive, NICU
7	31	+	20	20	0	84 (3)	84	−	+	34	2,167	AW
8	31	+	89	27	1	87 (7)	83	−	−	34	2,342	Alive CP
9	29	+	45.5	38.5	3	53 (7)	53	−	+	33	1,578	AW
10	28	+	39	39	0, 3 a	59 (3)	46	−	+	30	1,425	NICU; ID

Abbreviations: AW, alive and well; CP, cerebral palsy; FD, fetal death; ID, infant death; ND, neonatal death; NICU, neonatal intensive care unit.

Note: Underlined values represent low UVFV (<50 mL/minute/kg). UVFV, umbilical venous flow volume; measuring UVFV before TAS and delivery was performed within 4 days.

a After the second TAS.

We detected four subgroups dividing by UVFV/kg before and after TAS:

1. Normal UVFV before TAS–normal UVFV after TAS (normal–normal).
2. Low UVFV before TAS–normal UVFV after TAS (hypo–normal).
3. Normal UVFV before TAS–low UVFV after TAS (normal–hypo).
4. Low UVFV before TAS–low UVFV after TAS (hypo–hypo).

We found 12 cases of normal UVFVs both before and after TAS, and 75% (9/12) survived finally. They did not show low UVFV during the whole management period, suggesting subgroup 1. In the other 10 cases, they showed low UVFV at least one time during management.

Two cases of low UVFV before TAS showed normal UFVF after TAS and before delivery, suggesting subgroup 2 (cases 7 and 9). Their babies' prognoses were good and alive and well. TAS seemed to recover its hypovolemic condition. Four cases of normal UVFV before TAS showed low UFVF after TAS and before delivery, suggesting subgroup 3 (cases 1–4). Their prognoses were poor (one fetal death and three neonatal deaths). TAS could not recover and worsened the condition. Two cases showed low UVFV before TAS and low UVFV after TAS and before delivery, suggesting subgroup 4 (cases 6 and 10). One died neonatally, and since we decided on immediate delivery, one survived but needed long intensive care at the NICU. We regarded this group as subgroup 4. In one case of 8, post-day of TAS at 7 days, their UVFV was finally recovered and could extend the pregnant period and deliver a baby at 34 weeks' gestation, but the baby showed cerebral palsy at 3-year follow-up.

Among these low UVFV 10 cases, 1 fetal death, 3 neonatal deaths, 1 infantile death, and 1 case of cerebral palsy at 3-year follow-up occurred. Only two cases survived without a major handicap in this lower (at least on one occasion) UVFV group. In regard to the low UVFV in seven cases just before delivery, five cases expired and one survived after long-term NICU management (survival rate: 29%); in the other normal UVFV 15 cases, 11 survived (survival rate: 73%; Tables 3 and 4 ).

**Table 3:** Prognosis of thoraco-amniotic shunting cases defined by umbilical venous flow volume (cutoff: 50 mL/minute/kg) before delivery

	Before delivery, low UVFV (collapse)	Before delivery, normal UVFV (control)	*p* -Value
No. of cases	7	15	
Mean value of UVFV (SD)	19.5 (7.9)	198 (108)	
Median	18	184	0.0005
Range	11–34	59–405	
UAEDV at hypo UVFV	5/7 (71%)	2/15 (13%)	0.006
DV reverse flow of A wave	0/7	0/15	
Survival rate	2/7 (29%)	11/15 (73%)	0.074
FD	1	1	
ND + ID	4	3	
NICU > 84 d	1	2	
FD, ND, NICU > 84 d	7/7 (100%)	6/15 (40%)	0.017

Abbreviations: before TAS and delivery, findings confirmed within 4 days, respectively; FD, fetal death; ID, infant death; low, < 50 mL/minute/kg; ND, neonatal death; NICU, managed> 84 days; UVFV, umbilical venous flow volume (mL/minute/kg).

Note: UA-EDV; Umbilical artery absent end-diastolic velocity, survival rate, at least 6 months. The Fisher probability test and Mann–Whitney *U* test were used for statistical analysis. A *p* -value < 0.05 was considered significant.

**Table 4:** Final outcome of each subgroup, divided by UVFV/kg using a cut-off of 50 mL/minute/kg

Stage	Subgroup	*n*	Survival	Edema	Final
	Before TAS → after TAS		28 d	Improved	Survival
1	Normal → normal	12	9 (75%)	9/12 (75%)	9 (75%)
2	Hypo → normal	3	3 (100%)	3/3 (100%)	2 (67%)
3	Normal → hypo	4	0 (0%)	2/4 (50%)	0 (0%)
4	Hypo → hypo	3	2 (67%)	2/3 (67%)	1 (33%)

Note: Normal, over 50 mL/minute/kg; hypo, below 50 mL/minute/kg; before and after thoraco-amniotic shunting for severe fetal pleural effusion. Hypo UVFV/kg is defined below 50 mL/minute/kg. We divided four groups hypothetically.

To clarify the characteristics of the circulation, other parameters were evaluated when showing a decrease in fetal UVFV. Fetal skin edema resolved in 7 of 10 cases, and 8 of 10 showed absent umbilical artery end diastolic velocity (UAEDV) when measured at a low UVFV level at the same time examination. Ratio of UAEDV with low UVFV was detected with significant differences ( *p* = 0.006) in 5 of 7 (71%) versus 2 of 15 (13% as normal UVFV) cases. A total of 72 ultrasound examinations were performed on 22 cases. We divided the low and normal UVFV subgroups and compared cardiac function. In the low UVFV groups, a significantly lower Descending aorta- *V*_max_ (peak systolic velocity with angle correction within 60 degrees) 79.5 cm/sec, smaller heart size (CTAR) 19%, and UAEDV 61% were found, but DV reversal flow was not detected in both groups ( Table 5 ).

**Table 5:** Characteristics of fetal cardiac function by ultrasonographic evaluation compared with that of UVFV in fetal pleural effusion in 22 cases with 72 measurements

	Low UVFV/kg	Normal UVFV/kg	Statistics
Measure, *n*	18	54	
Exam; GA (wk)	30.4 (± 1.6)	29.4 (± 3.0)	NS
UVFV/kg (mL/min/kg)	32.5 (11–45.5)	108 (53–708)	<0.001
Ao- *V*_max_ (cm/s)	79.5 (± 21.9)	99.0 (± 19.7)	0.0015
CTAR	19.0 (4.5)	22.2 (± 5.6)	0.018
UA-AEDV	11/18 (61%)	6/54 (11%)	<0.001
MCA-PSV (cm/s)	36.6 (± 9.9)	40.5 (± 11)	NS
DV reverse	0/18	0/54	NS

Abbreviations: Ao- *V*_max_ , descending aorta maximum velocity corrected by angle within 60 degrees; CTAR, cardio-thoracic area ratio; DV reverse, ductus venosus reverse flow of A wave; low, < 50 mL/minute/kg; MCA-PSV, middle cerebral artery peak systolic velocity; UA-AEDV, umbilical artery-absent end-diastolic velocities; UVFV, umbilical venous flow volume (mL/minute/kg).

Note: Fisher's probability test, student's *t* , and Mann–Whitney *U* test were used for statistical analysis. A *p* -value < 0.05 was considered significant.

## Discussion

Recently, in Japan, the safety and effectiveness of TAS using a double basket catheter (Hakko, Nagano, Japan) was confirmed by a prospective study. 6 However, in some of our cases, even if skin edema resolved, the prognosis was poor. The reason this occurred was not apparent. One possible explanation is a fetal complication, such as a structural, systemic abnormality we could not detect prenatally. Another might be the situation of fetal cardiac collapse due to the combined factors of low UVFV, UAEDV, small heart, which we described in this analysis. We deemed the disappearance of skin edema to be a positive prognostic sign for survival; however, our analysis revealed that 70% of low UVFV cases showed complete resolution of skin edema, including cases with a poor prognosis. This phenomenon strongly suggests that the resolution of skin edema is not necessarily a good predictor of survival in the most severe cases. After a shunting procedure, fetal fluid leakage into the amniotic fluid occurs; however, with good placental function, enough fluid replacement can occur. This results in the maintenance of adequate fetal blood flow and cardiac output, and we can consider these cases to be successful fetal intervention cases. However, when placental function is compromised, for example, in cases of low fetal albuminemia and an edematous placenta of hydrops fetalis due to excess lymphatic fluid leakage to the thoracic cavity, maintenance of fluid balance is compromised. Under these circumstances, fetal fluid in third spaces such as the thoracic cavity and skin appears to resolve, but the fetal blood flow might be reduced, showing “intravascular dehydration.” For example, UAEDV and low descending aorta *V*_max_ flow are highly accurate for the measurement of a low UVFV with a significantly small CTAR ratio. These results suggest a low UVFV without adequate placental replacement. This phenomenon might reveal one kind of irreversible placental dysfunction.

Interestingly, DV A wave reverse flow was not detected in any of the cases. DV reverse flow entails an imbalance between UVFV (preload) and atrial volume, 15 acid–base status 16 in fetal growth restriction (FGR) and cardiac failure 17 18 due to fetal cardiac structural anomalies. A severe hypovolemic state (low afterload) might not manifest as a significant difference in UVFV (preload) and atrial volume, and would not result in reverse flow of the DV. This situation also represents a fetal hypovolemic status. In other words, parameters such as the DV wave are not adequate for evaluating hypovolemic status. In case 2 ( Table 2 ), just after a cesarean section for an extremely low UVFV, the infant expired in the NICU due to ineffective cardiac contractility and output. Considering these cases, we defined this situation as typical “fetal cardiac collapse.” As Frank–Starling's law says, if the fetal preload reduces too much because of reduced UVFV, fetal heart failure, such as contractility, would be affected.

After analyzing the longitudinal change of UVFV before and after TAS, we found four subgroups categorized as described below hypothetically ( Tables 4 and 6 ).

**Table 6:** A new criterion of fetal cardiac collapsing status and recommended management policy judging from umbilical venous flow volume/kg.

Stage	Subgroup	Pathopysiology	Management policy
1	Normal–normal	“TAS effective”	Extend the pregnancy period
2	Hypo–normal	“TAS; effective” hypo UVFV by transient small heart with enough compensation from the placenta	Extend the pregnancy period
3	Normal–hypo	“Collapsing” too much lymph fluid drainage without enough compensation from the placenta	Preparing for delivery, consulting with NICU management
4	Hypo–hypo	“Collapsed” collapse without any recovery and without enough compensation from the placenta	Immediate delivery to prevent fetal death, information to NICU management

Abbreviations: NICU, neonatal intensive care unit; TAS, thoraco-amniotic shunting; UVFV, umbilical venous blood flow volume.

Note: Cut-off 50 mL/minute/kg before and after thoraco-amniotic shunting for severe fetal pleural effusion. Normal: UVFV > 50 mL/minute/kg, hypo: UVFV < 50 mL/minute/kg.

Group 1: Normal UVFV before TAS and normal UVFV maintained until delivery. This group comprised 12 cases. The survival rate was 75%. When UVFV is maintained at a normal level, we can safely extend the pregnancy duration.
Group 2: Low UVFV improved after TAS. This group had a 67% survival rate. These fetuses merely exhibited that the high pressure by thoracic massive fluid pooling and UV inflow from a healthy placenta would be transiently disturbed physically; TAS would effectively resolve the high pressure; and enough cardiac dilatation and placental fluid replacement would begin after TAS. When UVFV is maintained at a normal level, we can also safely extend the duration of the pregnancy.
Group 3: UVFV was reduced to be low level after TAS for a period of time. In this group, excess fluid pooling would probably result in acute deterioration, and there might be inadequate and nonplacental fluid replacement. We consider this stage as “collapsing.” We should prepare for delivery if we detect a gradual deterioration of UVFV. Fetal death can be prevented with meticulous fetal monitoring.
Group 4: Low UVFV not improved by TAS at all. Placental replacement might already be compromised irreversibly, and fetal cardiac status might be “collapsed.” We should consider immediate delivery to prevent fetal death.

These categories would help the clinician to monitor the fetuses after TAS. Groups 1 and 2 would keep us during the pregnancy period with safety, and groups 3 and 4 would make us starting preparing for early delivery.

Our study has some limitations in regard to the analysis of fetal survival. In some cases, there may be another lethal anomaly that could not be diagnosed prenatally that would influence the prognosis. 7 Therefore, the chart we developed cannot determine the prognosis in all cases. This chart would merely contribute to diagnosing fetal circulations, such as homeostasis and fluid balance. Furthermore, in this complicated situation of a nonhomogeneous population, a precise analysis of TAS effectiveness is compromised. In predicting the final prognosis of each case by circulatory parameters, we should incorporate this new classification.

The methodology of measuring UVFV has been discussed. 10 11 We selected an intrahepatic portion rather than a free loop of umbilical in deference to our skill set pertaining to this type of analysis. Using a hepatic portion has been reported to have high reproducibility; Spanish researchers showed a higher reproducibility when using a hepatic portion compared with that of the umbilical arterial pulsatility index. This study encourages us to continue to use this parameter.

We set a transient cutoff value for low UVFV at 50 mL/minute/kg hypothetically. This value appeared to lie at the 2.5th percentile of another study. 9 If we accrue more cases in the future, we will conduct a receiver operating curve analysis. The foregoing is also a limitation of this study; however, for mild FGR cases during the late pregnancy period, 68 mL/minute/kg is a critical cutoff value for assessing fetal asphyxia. 19 As such, the level of our cutoff value appears to be appropriate.

We also speculated fetal cardiac collapse using UVFV and UA flow pattern due to its convenience, but ideally, it would be better to use additional parameters such as combined cardiac output (CCO). 20 Though the CCO has some variability in its values, these data would be helpful to analyze the pathophysiology of fetal cardiac collapse in the future.

In conclusion, our new grouping of the evaluation of fetal collapsing situations could serve as a new assessment tool to precisely evaluate fetal and placental status in TAS for severe pleural effusion.

## Notes

**Corrigendum:** This article was corrected as detailed in the corrigendum published on September 26, 2025 (doi: 10.1055/a-2706-6895).
