# Corrigendum: Fetal Cardiac Collapse Diagnosed By Umbilical Venous Flow Volume After Thoraco-Amniotic Shunting for Severe Pleural Effusion

**DOI:** 10.1055/a-2706-6895

**Published:** 2025-09-26

**Authors:** Yuichiro Takahashi, Shigenori Iwagaki, Kazuhiko Asai, Masako Matsui, Ryuichi Shimaoka, Hitomi Ono, Saki Inuzuka

**Affiliations:** 1 Department of Fetal-Maternal Medicine, Obstetrics, Gifu Prefectural General Medical Center, Gifu City, Gifu, Japan; 2 Department of Fetal-Maternal Medicine, Nagara Medical Center, Gifu, Japan

It has been brought to the publisher's attention that in the above published article in *American Journal of Perinatology Reports* , Volume 15, Issue 3, September 16, 2025 (doi:  ), reference 20 was incorrect and should be replaced with the following citation:

Kiserud T, Ebbing C, Kessler J, Rasmussen S. Fetal cardiac output, distribution to the placenta and impact of placental compromise. Ultrasound Obstet Gynecol 2006;28(02):126-136

This article has been corrected online.

